# Integration of services for older people: back to funding

**Published:** 2012-09-04

**Authors:** Réjean Hébert

**Affiliations:** Université de Sherbrooke, Canada

**Keywords:** coordination, case-management, funding, long-term care insurance

## Abstract

**Purpose:**

To demonstrate that funding is an essential component of integrated care, even in a universal publicly funded health care system.

**Context:**

The PRISMA coordination-type model is actually being generalized in the Province of Québec (Canada) concurrently with the creation of Health and Social Services Centers (HSSC), a merging of hospitals, community centers and nursing homes in a local area.

**Case description:**

PRISMA originally included six components: coordination tables, single entry point, case management, individualized service plan, unique assessment tool and computerized clinical chart. Funding was not included since it was hypothesized as not necessary in the context of a universal publicly funded Beveridge-type health care system. The creation of HSSC implies that all public institutions are under the same governance structure with budget responsibilities. In addition to slowing down the implementation process of PRISMA, this structural integration also marginalized home care in the financial distribution, hospitals and nursing homes being seen as priorities. This has limited the availability of home care services, reducing the case-managers’ power to get the appropriate services to their patients.

**Discussion and conclusion:**

The introduction of a specific funding for long-term care, like the long-term care insurances implemented in many European and Asian countries would give the case-managers a better lever to provide their patients with the necessary services to remain at home. Funding should then be added as a 7th component of the PRISMA model.

Powerpoint presentation available at http://www.integratedcare.org at congresses – San Marino – programme.

